# Supplementary material for: SlZIP11 mediates zinc accumulation and sugar storage in tomato fruits
Source: PeerJ. 2024 May 29;12:e17473. doi: 10.7717/peerj.17473 (PMC11143971; doi:10.7717/peerj.17473)
Supplement: Supplemental Information 1 — IMG_10DPA, immature green fruits (10 day post-anthesis); MG_35DPA, mature green fruits (35DPA); Breaker_38DPA, breaker fruits (38DPA); Orange_41DPA, orange fruits (41DPA); Red_44DPA, red ripen fruits (44DPA). The data was obtained from the tomato database (https://solgenomics.net/) [file peerj-12-17473-s001.docx]

**Supplemental Table S1. Summary of primer sequence**

| Name of primer | Sequence |
| --- | --- |
| qACTIN-F | TGTCCCTATTTACGAGGGTTATGC |
| qACTIN-R | AGTTAAATCACGACCAGCAAGAT |
| qSlSWEET7a-F | TGATGCCTACATTCTCGCACC |
| qSlSWEET7a-R | TCCTTTAGCCTCTCTTGCTGCC |
| qSlZIP11-F | GCATCTTATTGATCGTTGCACT |
| qSlZIP11-R | ACTTACAGTCCAAAGAGCTCTC |
| STE-SlSWEET7a-F | ATTAACAAGGCCATTACGGCCACTTTTAATAGGGACAATGCTAGGTTTG |
| STE-SlSWEET7a-R | AACTGATTGGCCGAGGCGGCCCCAACTCTAAAATCACTTCTCGGGTTTTG |
| GFP-SlZIP11-F | CGGGGGACGAGCTCGGTACCATGGCACGTTTACACTTGTTTCTTG |
| GFP-SlZIP11-R | CTAGAGGATCCCCGGGTACCAGTGTCCCATATCATCACCACAGC |
| GWSlZIP11-F | GGGGACAAGTTTGTACAAAAAAGCAGGCTTCGGTACCATGGCACGTTTACACTTGTTTCTTG |
| GWSZIP11-R | GGGGACCACTTTGTACAAGAAAGCTGGGTGACTAGTAGTGTCCCATATCATCACCACAGC |
| OESlZIP11-F | GCTCTAGAATGGCACGTTTACACTTGTTTCTTG |
| OESlZIP11-R | CCCCCGGGAGTGTCCCATATCATCACCACAGC |
| TRV-SlZIP11-F | CCTCCATGGGGATCCGGTACCATGGCACGTTTACACTTGTTTCTTG |
| TRV-SlZIP11-R | GAGACGCGTGAGCTCGGTACCCGCAGTTCCCAAGAAAACCC |


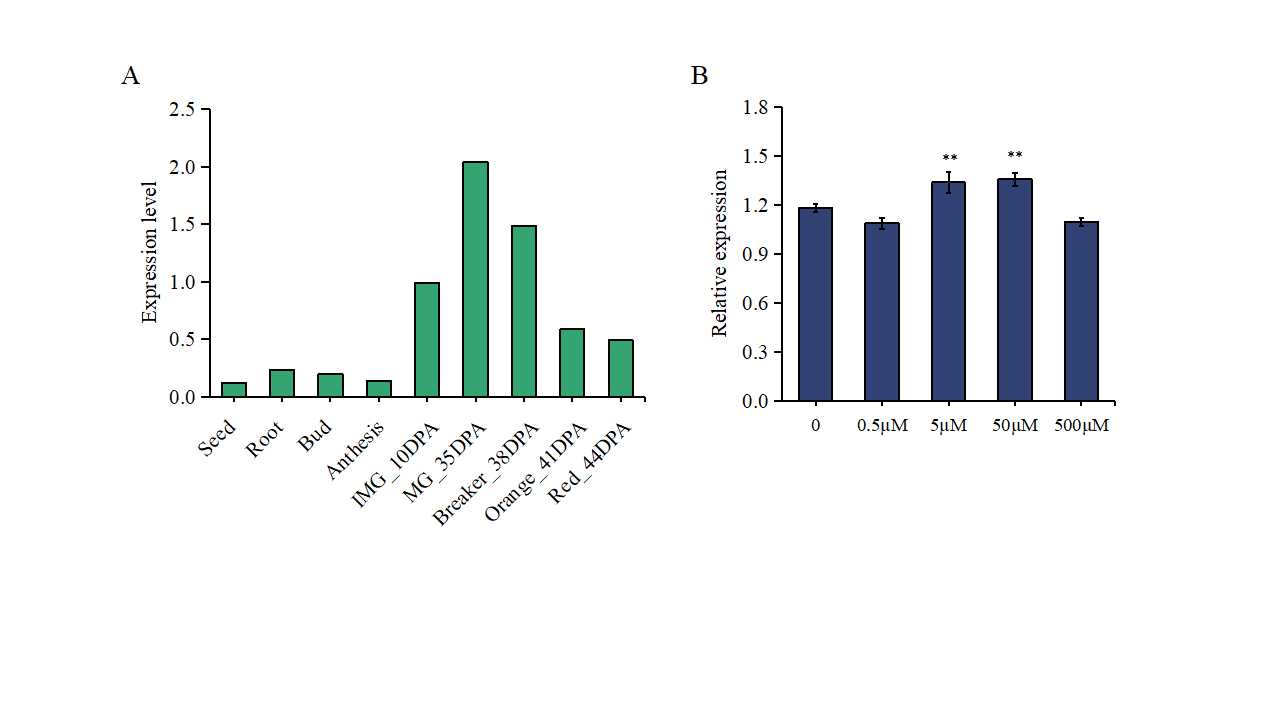


**Supplemental Figure S1. Expression level of SlZIP11 in ‘Heinz 1706’.**

IMG_10DPA, immature green fruits (10 day post-anthesis); MG_35DPA, mature green fruits (35DPA); Breaker_38DPA, breaker fruits (38DPA); Orange_41DPA, orange fruits (41DPA); Red_44DPA, red ripen fruits (44DPA). The data was obtained in the tomato database (https://solgenomics.net/).
